# A qualitative study of the promotion of exclusive breastfeeding by health professionals in Niamey, Niger

**DOI:** 10.1186/1746-4358-5-8

**Published:** 2010-08-08

**Authors:** Aïssata Moussa Abba, Maria De Koninck, Anne-Marie Hamelin

**Affiliations:** 1 Programme interfacultaire de doctorat en santé communautaire, Faculté des sciences infirmières et Faculté de médecine, Université Laval, Québec City, Canada; 2 BP 13210 Niamey, Niger; 3 Department of Social and Preventive Medicine, Faculty of Medicine, Université Laval, Québec City, Canada; 4 Department of Food Sciences and Nutrition, Faculty of Agriculture and Food Sciences, Université Laval, Québec City, Canada

## Abstract

**Background:**

The practice of exclusive breastfeeding depends on various factors related to both mothers and their environment, including the services delivered by health professionals. It is known that support and counseling by health professionals can improve rates, early initiation and total duration of breastfeeding, particularly exclusive breastfeeding. Mothers' decisions are influenced by health professionals' advice. However, in Niger the practice of exclusive breastfeeding is almost non-existent.

The purpose of this exploratory study, of which some results are presented here, was to document health professionals' attitudes and practices with regard to exclusive breastfeeding promotion in hospital settings in the urban community of Niamey, Niger.

**Methods:**

Fieldwork was conducted in Niamey, Niger. A qualitative approach was employed. Health professionals' practices were observed in a sample of frontline public healthcare facilities.

**Results:**

The field observation results presented here indicate that exclusive breastfeeding is not promoted in healthcare facilities because the health professionals do not encourage it and their practices are inappropriate. Some still have limited knowledge or are misinformed about this practice or do not believe in it. They do not systematically discuss exclusive breastfeeding with mothers, or they mention it only briefly and without giving any explanation. Worse still, some encourage the use of breast milk substitutes, which are frequently promoted in healthcare facilities. Thus mothers often receive contradictory messages.

**Conclusion:**

The results suggest the need to train or retrain health professionals with regard to exclusive breastfeeding, and regularly supervise their activities.

## Background

The WHO and UNICEF jointly recommend that women exclusively breastfeed their infants for the first six months and continue to breastfeed into the second year of life or longer. This feeding method is the normative model [1]. The importance of breastfeeding, especially exclusive breastfeeding (EBF), is well established for the infant, the mother and the family [2-8] and there are risks of not breastfeeding infants, particularly in poorer environments where social, economic and unsafe hygienic conditions increase the risk of infections and undernourishment. In those settings when infant formula are used, they are introduced early and over-diluted. In Niger, nearly all mothers start breastfeeding and continue until 21 months on average, but only 1% of infants are exclusively breastfed for the first six months, and this rate did not change between 1992 and 2005 [9-11].

EBF is rare for a variety of reasons related to both mothers and their environment, including services delivered by health professionals (HP) [12]. Some studies have shown that HPs play an important role in the mothers' decisions and practices [13,14]. It is well known that support and counseling from health care providers can improve breastfeeding rates as well as early initiation and total duration of breastfeeding, and make mothers more confident [14-20]. Some studies found that mothers often report being given incorrect or incomplete information, or that physicians were apathetic about breastfeeding during medical visits [13,14,21,22]. Other studies also showed a general lack of breastfeeding knowledge among HPs (nurses, medical assistants, physicians, pediatricians) [23-25]. No study has looked at these aspects of EBF promotion among HPs in Niger. Thus, the aim of this study was to explore HPs' attitudes and practices with regard to EBF promotion in hospital settings in the urban community of Niamey, Niger. More specifically, the objectives were to document HPs' attitudes and practices with regard to EBF promotion, to identify those that might hinder increased EBF rates in Niger, and to suggest interventions that could help improve the situation.

## Methods

We based our approach on Lutter's theoretical model, which identifies three levels of determinants (individual, family and society) that influence feeding practices, including breastfeeding, and looked specifically at the level of society, which is the level at which the author places attitudes and medical norms [12]. Our observations focused on the dimensions found in previous research on this determinant in other settings. These studies indicated that sociodemographic variables and knowledge would influence HPs' attitudes, which in turn motivate them and determine their practices. These practices are also influenced by available resources and working conditions [20,26-28].

### Study design

Given the lack of data on this subject, our study was exploratory and employed a qualitative approach. Two data collection techniques were used. In the first phase, we made direct observations in a sample of three healthcare facilities (HF) in Niamey, and in the second phase, discussion groups were organized with 43 volunteer HPs recruited in a sample of twelve HFs in Niamey. The findings from these discussion groups are the subject of another publication [29]. This article reports the data collected through observing HPs' practices and their working conditions. We also systematically paid attention to the interactions between HPs and mothers to determine 1) if EBF was discussed, 2) if explanations and advice were given, and 3) if the context reflected the HFs' official position that EBF must be promoted. One of the indicators of this last dimension was the attention given to infant formula.

The observations were conducted overtly by the first author of this article. They were done initially, before the focus groups, in order to avoid introducing a bias such as HPs trying to modify their usual practices after discussions on desirable attitudes or behaviors. The observations took place within working hours, generally from 8 am to 2-3 pm every day for 82 days from 7 January to 28 March 2008 in a maternity hospital, an integrated health center (CSI) type 1 [a first line health center which serves 5,000 residents] and a CSI type 2 [a first line health center which serves 5,000 to 15,000 residents and has 5 to 10 beds for observation], which are frontline public HFs used by most of the Niamey population. The three HFs in the sample were randomly chosen from the 37 HFs in the three districts of Niamey.

### Data collection tools and analysis

The conceptual framework guided the construction of an observation grid used to record information about the general situation in the HFs and the practices of the HPs. It covered the dimensions mentioned above and was used to compile information regarding the advice and explanations given to mothers about feeding infants, techniques for expressing and preserving milk, assistance with putting the baby to the breast and the duration of interactions. During the observations, informal discussions also took place with some practitioners and mothers. A journal was kept to record the notes presented in this article. Data analysis was conducted manually. The content of the recorded material was analyzed following the steps described by Mayer and Deslauriers [30]. First, the notes were entered for each HF. Then, using an inductive approach, the notes were coded for the meanings found and grouped into subthemes, from which themes were identified. These themes were added to those from the observation grid and used for the different analyses.

### Subject selection and ethical considerations

All 31 HPs working in the HFs in the sample were eligible since the only criterion was to be employed in one of the facility included in the sample. Indeed, all HPs working in frontline health structures, whatever their characteristics (age, school level, experience etc.) are in regular contact with pregnant or postpartum women and are supposed to take any opportunities to promote exclusive breastfeeding. A pamphlet introducing the researcher and describing the nature and procedures of the study, management of the data and the dissemination of results, as well as the personal benefits, risks and rights related to participating, were given to all the HPs observed. Written consent was obtained from each participant and a commitment was made to safeguard confidentiality and anonymity. The study was approved by the Research Ethics Committee of Université Laval in Québec City, Canada and authorized by the public health department in Niger.

## Results and discussion

Several studies have shown that support from HPs improves breastfeeding rates in general and EBF in particular [15-17,20]. However, according to our observations, this support is non-existent or inadequate in some Niamey HFs. EBF promotion does not seem to be a priority for the HPs concerned.

### Context and resources of the healthcare facilities

Our observations took place during various types of consultation where pregnant women, women giving birth and mothers of infants up to six months of age are seen, and also consultations involving vaccinations, healthy and sick infants.

#### Physical environment

Generally speaking, the internal organization and certain practices in the HFs were not conducive to promoting EBF effectively. Access to the premises was not controlled. We witnessed purely commercial activities unrelated to health care, such as auction sales. These activities went on during consultations, which distracted the mothers and increased the noise level, making discussions between mothers and professionals difficult [31]. Often mothers entered the children's consulting room in groups of two or three and there were no baby changing tables or chairs for them to use. Such conditions were not at all conducive to being able to promote EBF effectively. In-depth one-on-one interviews to give advice to each mother were difficult to do and often done hastily. On the other hand, there were certain practices that should be maintained and encouraged in the HFs, such as the absence of nurseries, as recommended by the WHO [32]. Babies stayed in the same room as their mother, which encourages mother-child interaction and the initiation and practice of on-demand feeding. In addition, HPs took away from the mothers all visible equipment used for mixed feeding, such as cups and baby bottles. However, HPs did not explain the reasons for these actions to the mothers and particularly the dangers of not exclusively breastfeeding, including the fact that the infant would not benefit from the antibodies in the mother's milk and be more vulnerable to infections such as acute respiratory infections and diarrhea diseases [1,3,5], which are some of the leading causes of infant mortality in Niger [33].

#### Human and material resources

Niamey HFs have to deal with staff shortages and difficult physical conditions. HP workforce levels in Niger do not meet WHO standards. As one midwife explained: *"We are overwhelmed to the point of asking volunteers and trainees to help because sometimes we have as many as 450 deliveries per month*." She added that: *"Working conditions are very difficult. There is absolutely no disposable equipment. Everything is reusable and used again after disinfecting it with bleach. The facility does not have a generator so every midwife has to carry a flashlight to use during power cuts. If the water is cut off, the guard is the only person with a key to access the water tank"*.

Even though HPs followed certain practices recommended to encourage breastfeeding, such as immediate skin-to-skin contact after delivery, they did not offer any assistance with initiating breastfeeding in the first half hour after birth. They were more concerned with disinfecting the equipment and caring for the mothers (sutures, for example) and babies (e.g., measuring them) as well as women in labour. During all our observations, we never witnessed HPs providing practical sessions for mothers, such as showing them how to put the baby to the breast. The only situation we observed where EBF was explained in detail and putting the baby to the breast was checked and corrected involved a "femme relais" (FR) [femmes relais are women residing in the districts where the healthcare facility is. They are usually older women who have experience with childbirth and infant health and know most of the women of child-bearing age in the area they cover. They are volunteers who are trained by health professionals to educate the population on certain health-related matters. Their number varies from district to district]. Some HPs maintained that they did not have enough time to promote EBF. They said that each day they see "*between 30 and 50 pregnant women just for prenatal checkups, not counting those who come for family planning*" [midwife]. Also, in cases involving infants less than six months old suffering from diarrhea, the HPs were concerned with treating the diarrhea without thinking to ask about infant feeding method.

#### A valuable resource: "femmes relais"

The "femmes relais" are an important resource in promoting EBF, as was stressed by the HPs in the HFs observed and the discussion group participants. According to one nurse, four FRs were available to her HF, but did not really do much except on national vaccination days (NVD) for which they receive a bonus. Normally, they did things like cooking demonstrations, breastfeeding awareness, neighbourhood population mobilization during vaccination campaigns, and looking for women who stopped going to the HF. This HP added that: *"they are very effective and know how to mobilize the women for all aspects, but they need a little bonus so they are more motivated*" [nurse]. In fact, during our observations, the only time we witnessed detailed reasons given for recommending EBF was during a health education session conducted by an FR. She explained some of the benefits and practical aspects. She advised against bottle feeding and stressed its many disadvantages. This FR also demonstrated the correct position for breastfeeding, then asked each mother to put her baby to her breast so that she could identify and correct incorrect positioning. As the discussion group participants pointed out, this example suggests that it would be beneficial to organize and train FRs as a resource to do promotional activities and help reduce the HPs' workload. In an informal discussion, one FR said: *"We currently participate in the final health education session [before mothers leave the maternity ward] but not on a regular basis; we do not generally do home visits except in rare cases where we find pregnant women who do not come to prenatal checkups. However, we are frequently mobilized during NVD campaigns*."

#### Promotion of infant formula

Contrary to the International Code of Marketing of Breastmilk Substitutes, infant formula promoters [generally called medical delegates, they are pharmaceutical laboratories representatives who are in charge of doing the promotion of their products, which include medicines and breast milk substitutes] try to convince HPs of the need to use these products to feed infants. For example, concerning so-called "starter" milk designed for infants up to six months, one promoter explained to HPs that: *"Even if we stress breastfeeding, it is impossible to do without canned milk [this refers to tins of powdered infant formula], because some mothers have difficulty breastfeeding and others don't have enough milk, some can afford canned milk, the process of expressing breast milk is unpleasant; and furthermore, Healthmilk [fictitious name] contains essential amino acids that strengthen the baby's immune system; it is very similar to mother's milk*" [medical delegate]. A midwife supported these arguments, saying that "*it is important to help mothers feed their babies*" and adding "*even if our maternity ward is Baby-Friendly" *[midwife]. Another medical delegate came to promote *"drugs to stimulate appetite and control fever, vomiting and diarrhea." *He told HPs that "*they were designed for infants from one month old," *gave them samples and recommended that they prescribe these products. Promoting and prescribing these types of products encourage mothers to introduce foods as early as one month. Few posters showing breastfeeding mothers and recommending EBF up to 4-6 months could be seen in two of the three HFs we observed. However, there were others advertising infant formula, which inevitably discourages EBF. In one consulting room visited by about 70 mother/child dyads per day, there were no breastfeeding posters although a medical delegate had persuaded HPs to put up stickers advertising infant formula visible from the examination table. For example, when the mother of a healthy three-month-old who was growing well told one HP: *"I want to start giving him canned milk because I want to start going to the sewing room*," the professional did not talk to her about the option of expressing and preserving her breast milk, but prescribed baby "*Healthmilk"*, showing the mother the sticker and saying: *"This is good milk, you see the beautiful baby" *[social worker]. The HP told the mother to bring in the powdered infant formula so she could show her how to use it. With practices like these, HPs are encouraging mixed feeding and giving mothers the message that breast milk substitutes are also a good choice for infant nutrition and health.

Niger has subscribed to the international initiatives aiming at promoting, protecting and supporting breastfeeding which are mainly: the Code of Marketing of Breast-milk Substitutes, the Innocenti Declaration and more recently the Global Strategy for Infant and Young Child Feeding. Despite all this, the participants stated, and we also noticed during our observation sessions, that the Code of Marketing of Breast-milk Substitutes, to which Niger has subscribed, isn't taken into account because the promotion of these products is still done in the healthcare facilities since there is no strict control. This constitutes, according to them, an important obstacle to the promotion of exclusive breastfeeding. There were, in the same facility, contradictory messages which created confusion among mothers and even among certain HPs. Obviously, in this particular healthcare facility, it is difficult to achieve optimal food practices such as exclusive breastfeeding. Moreover, according to the evaluation made by UNICEF and the Ministry of Health of Niger, between 1996 and 2005, 36 healthcare facilities received accreditation as Baby Friendly Hospitals. However, in 2007 only 13 retained BFHI status; the others lost it because they didn't respect all the Ten Steps for Successful Breastfeeding and the International Code of Marketing of Breast-milk Substitutes [34].

### Practices of health professionals

#### Indifference or ignorance?

Health education group sessions given by the HFs on topics such as malaria, vaccination, prenatal checkups, prevention of mother-to-child transmission (MTCT) of HIV, etc., were usually organized by the HPs before the day's other activities. On average they lasted 15 minutes and did not include a question period. Some women did not understand the language in which they were given and there were no translation services. And EBF was rarely mentioned during these health education sessions or individual consultations. For example, out of 25 women seen in a day in one prenatal consulting room, breastfeeding was not discussed with any of them. Often HPs gave advice only to mothers who asked for it or had a particular problem, such as a low-weight baby. Our findings are similar to others reported in the literature [35]. During our observations, we noted that the most of the time (at least 60%) spent with each woman during prenatal checkups was devoted to MTCT counseling. Aspects related to mother and child nutrition were rarely mentioned. Mothers-to-be were not given any preparation, advice or explanations about feeding options. They were only encouraged to talk about their health problems and were not given the opportunity to ask questions about nutrition. In addition, depending on funding, HPs were motivated to stress one topic more than another. This source of motivation was confirmed by some of the discussion group participants; as one nurse said: *"For example MTCT is the biggest topic right now because that program is very well funded. There is very little funding for breastfeeding." *Funding is a very important aspect, because in Africa in general and in Niger in particular, HPs are paid to attend continuing education and retraining sessions.

With regard to practical help with breastfeeding, we did not see any HP provide this service during all our observations. Although some mothers used incorrect feeding positions in front of the HPs, the HPs did not intervene to help them. These HP behaviours might be attributable to certain obstacles that were brought up by the discussion group participants [29]. Lack of training was mentioned as the main obstacle to EBF promotion. During informal conversations, some HPs told us that during their academic training, they had only a two hour course about breastfeeding in general (not exclusive breastfeeding specifically) whereas the WHO recommends at least 18 hours of training including the practical and clinical aspects [36]. And, in the syllabus of the main schools/institutes/faculty of HPs' training that we consulted, there is no indication of a course about breastfeeding. In fact, other studies have found that HPs have limited knowledge of EBF [23-25]. The following conversation illustrates this: To the mother of a four-month-old who said she was bottle-feeding her baby powdered infant formula, a HP did not advise her against mixed or bottle feeding but said: "*Since he is four months old, you can also give him thin cereal and at six months you can start him on thick cereal" *[social worker]. To some mothers with infants under six months, the same HP advised giving orange juice. She told other mothers to give nothing but breast milk, but did not tell them why or until what age. With some mothers, she never asked how they were feeding their infants at all. Contradictory advice could be confusing, especially if the mothers talk to each other afterwards.

We also observed another factor discouraging EBF promotion, particularly in the maternity unit where mothers had their first contact with their newborns and were probably more willing to listen to advice about good practices for the baby's health. Their stay in the unit was very short, only 24 hours after delivery, which limits the contact between HPs and mothers and the time to promote EBF. During these 24 hours, women only saw the HPs if mother or baby has a specific health problem. On the other hand, advantage was not taken of other good opportunities to promote EBF, such as when mothers returned for the baby's TB vaccination (which usually occurs in the first week after childbirth), and could have been told that their milk contains antibodies that act like a vaccine.

#### Harmful beliefs

During one observation session, some HPs told us informally that *"it will be hard to practice EBF in Niger because of people's beliefs and especially during the hot season*" [social worker]. They themselves thought that "*water at least must be given*" [social worker]. These beliefs are detrimental to the promotion of EBF and also explain why HPs encouraged mothers to practice mixed feeding, as in the case of a mother of a three-month-old who said that she "*gives Nutrimilk [fictitious name] in addition to breast milk because the baby regurgitates; I think it's the breast milk that he can't keep down and that Nutrimilk stays in his stomach longer*." One HP asked a colleague to recommend *Healthmilk *(the medical delegate had visited that morning) or prescribe a special anti-regurgitation milk sold in the pharmacy. Rather than taking the opportunity to explain to the mother why this belief is unfounded and stop it from being repeated, she encouraged her to use infant formula. This lack of conviction on the part of the HPs seemed to predispose them not to promote EBF, and certainly not to act as leaders in their community.

#### Messages given to mothers: late, incomplete and contradictory

The verbal support given to mothers was often incomplete and sometimes the opposite of what is recommended. In the rare cases where HPs talked about EBF, they did not give any convincing explanation, as is shown in this advice given by an HP during a health education session: *"Don't give anything to the baby, not even water, until six months because mother's milk is 90% water" *[midwife]. The reasons for recommending EBF, its benefits and the disadvantages of mixed feeding or completely avoiding breastfeeding were not explained. Colostrum was also rarely mentioned; when it was, mothers were simply told not to throw it away, without any explanation of its role, its importance and the mistaken beliefs about it. Because mothers are usually very motivated to comply with the vaccination schedule, this could be used as an opportunity to explain that colostrum acts as the first vaccine and contains substances that protect the baby. For example, during a one-on-one consultation, one HP counseled a pregnant woman as follows: *"You must eat until you are satiated so that you are strong enough to push during the delivery; you must also give your first milk to the baby to protect it against disease and not give anything except breast milk for the first six months" *[midwife]. However, advising a mother to practice EBF without giving reasons, especially in view of the beliefs prevalent in Niger, is not enough to convince her and does not give her enough information to pass on to her family. The nutritional advice HPs gave was more concerned with supplementary foods that should only be started at the age of six months [37-40]. Besides, HPs often asked the question: *"What does the baby eat?" *to which the great majority of mothers replied, "*cereal*" and HPs added: *"With breast milk, right?*" This suggests that introducing cereal is a good idea. In addition, even mothers who may not be breastfeeding felt obliged to say they were. Also, some HPs continued to repeat the advice given in the child's health booklet, which completely contradicts the EBF recommendation. This official document for monitoring the infant's health and development is out-of-date but is still used in HFs and is a major source of confusion in the information it purveys, especially for mothers who can read.

Our study has several limitations since it does not cover all possible factors for EBF rate stagnation in Niger. First, the recruitment of the participants was on a voluntary basis and the health professionals may have changed their practices since they were being observed. Second, we have not explored in depth some dimensions such as: HPs' knowledge, their beliefs and the mothers' opinions about the EBF promotion, the attitudes and practices of HPs. Third, the results have not been validated with the participants and cannot be generalized as they could be different in the other regions of Niger. Finally, there could be some limitations of, and criticisms leveled against content analysis in qualitative research, including the subjectivity of the researcher [30].

## Conclusion

Despite the limitations mentioned above, we were able to identify and describe the practices of HPs. It must also be underlined that no such field study has been done before in Niger. The field observations showed that in general HPs rarely promote EBF in hospital settings and their practices are inappropriate. They do not discuss EBF systematically. They rarely think of recommending that mothers of infants under six months give only breast milk, sometimes because of a lack of knowledge and time. Most importantly, they never take the time to do any real EBF promotion by clearly explaining to mothers the reasons for and benefits of this recommendation, and also informing them of the risks related to mixed feeding. Worse still, some HPs do not believe in EBF and encourage the use of breast milk substitutes. Promoting these products in healthcare facilities is a real obstacle to the promotion of EBF and could be a source of confusion for mothers making a decision. However, given the traditional Nigerien practice of introducing different kinds of liquids to newborns right after birth, it is very difficult for mothers to choose to exclusively breastfeed their infants when they are getting contradictory messages or not getting the explanations they need to convince them of the benefits. Our results indicate that it is absolutely vital to train, retrain and inform HPs and supervise their practices regularly. In addition, FRs are a resource that should be developed and organized because they could play an important role in promoting EBF, which would help improve EBF rates.

## Competing interests

The authors declare that they have no competing interests.

## Authors' contributions

AMA carried out the study including the conception and design, data collection, analysis and interpretation, and wrote the manuscript. MDK was the supervisor of AMA for her doctoral thesis and worked closely with her in preparing the study proposal, supervising the data collection and analysis, and revising the manuscript. AMH was the co-supervisor and worked with AMA and MDK in preparing the study proposal and revising the manuscript. All authors read and approved the final manuscript.

## References

[B1] AAP (American Academy of Pediatrics) Breastfeeding and the use of human milk: policy statement Pediatrics 2005 115 2 496 506 10.1542/peds.2004-2491 15687461

[B2] PAHO (Pan American Health Organization) Breastfeeding IMCI Bulletin 2002 7

[B3] Feachem RG Koblinsky MA Interventions for the control of diarrhea diseases among young children: promotion of breast-feeding Bull World Health Organ 1984 62 271 291 6610496 PMC2536296

[B4] Victora CG Smith PG Vaughan JP Nobre LC Lombardi C Teixeira AM Fuchs SM Moreira LB Gigante LP Barros FC Evidence for protection by breast-feeding against infant deaths from infectious diseases in Brazil Lancet 1987 2 319 322 10.1016/S0140-6736(87)90902-0 2886775

[B5] Huttly SRA Morris SS Pisani V Prevention of diarrhoea in young children in developing countries Bull World Health Organ 1997 75 163 174 9185369 PMC2486931

[B6] Kramer MS Chalmers B Hodnett ED Sevkovskaya Z Dzikovich I Shapiro S Collet JP Vanilovich I Mezen I Ducruet T Shishko G Zubovich V Mknuik D Gluchanina E Dombrovskiy V Ustinovitch A Kot T Bogdanovich N Ovchinikova L Helsing E Promotion of breastfeeding intervention trial (PROBIT): a randomized trial in Republic of Belarus JAMA 2001 285 4 413 420 10.1001/jama.285.4.413 11242425

[B7] Jones G Steketee RW Black RE Bhutta ZA Morris SS How many child deaths can we prevent this year? Lancet 2003 362 65 71 10.1016/S0140-6736(03)13811-1 12853204

[B8] Horta BL Bahl R Martines JC Victora CG Evidence on the long term effects of breastfeeding: Systematic reviews and meta-analysis World Health Organization 2007

[B9] Kourguéni IA Garba B Barrere B Enquête Démographique et de Santé 1992 1993 Direction de la Statistique et des Comptes Nationaux, Ministère des Finances et du Plan, Niamey, Niger

[B10] Attama S Seroussi M Kourguéni AI Koché H Barrère B Enquête Démographique et de Santé 1998 1999 Care International/Niger et Macro International Inc., Claverton, Maryland, USA

[B11] Bellamy C La Situation des Enfants dans le Monde 2005: l'Enfance en Péril 2004 UNICEF, New York

[B12] Lutter CK Breastfeeding promotion: Is its effectiveness supported by scientific evidence and global changes in breastfeeding behaviors? Adv Exp Med Biol 2000 478 355 368 11065085 10.1007/0-306-46830-1_30

[B13] Losch M Dungy C Russell D Dusdieker L Impact of attitudes on maternal decisions regarding infant feeding J Pediatr 1995 126 4 507 514 10.1016/S0022-3476(95)70342-X 7699527

[B14] Humenick S Hill P Spiegelberg P Breastfeeding and health professional encouragement J Hum Lact 1998 14 305 310 10.1177/089033449801400414 10205449

[B15] de Oliveira MI Camacho LA Tedstone AE Extending breast-feeding duration through primary care: a systematic review of prenatal and postnatal interventions J Hum Lact 2001 17 326 343 10.1177/089033440101700407 11847902

[B16] Sikorski J Renfrew MJ Pindoria S Wade A Support for breastfeeding mothers Cochrane Database Syst Rev 2002 CD001141 11869593 10.1002/14651858.CD001141

[B17] Guise JM Palda V Westhoff C Chan BKS Helfand M Lieu TA The effectiveness of primary care-based interventions to promote breastfeeding: systematic evidence review and meta-analysis for the US Preventive Services Task Force Ann Fam Med 2003 1 70 80 10.1370/afm.56 15040435 PMC1466575

[B18] Isabella PH Isabella RA Correlates of successful breastfeeding: a study of social and personal factors J Hum Lact 1994 10 257 264 10.1177/089033449401000421 7619281

[B19] Freed GL Clark SJ Sorenson J Curtis P National assessment of physicians' breast-feeding knowledge, attitudes, training, and experience JAMA 1995 273 6 472 476 10.1001/jama.273.6.472 7837365

[B20] Britton C McCormick FM Renfrew MJ Wade A King SE Support for breastfeeding mothers Cochrane Database Syst Rev 2007 CD001141 17253455 10.1002/14651858.CD001141.pub3

[B21] Coreil J Bryant CA Westover BJ Bailey D Health professionals and breastfeeding counseling: client and provider views J Hum Lact 1995 11 4 265 271 10.1177/089033449501100411 8634102

[B22] DiGirolamo AM Grummer-Strawn LM Fein SB Do perceived attitudes of physicians and hospital staff affect breastfeeding decisions? Birth 2003 30 2 94 100 10.1046/j.1523-536X.2003.00227.x 12752166

[B23] Larsen PG Effect of an educational intervention about breastfeeding on the knowledge, confidence, and behaviors of pediatric resident physicians Pediatrics 2002 110 5 e59 10.1542/peds.110.5.e59 12415065

[B24] Shah S Rollins NC Breastfeeding knowledge among health workers in rural South Africa J Trop Pediatr 2005 51 1 33 38 10.1093/tropej/fmh071 15601651

[B25] OlaOlorun FM Lawoyin TO Health workers' support for breastfeeding in Ibadan, Nigeria J Hum Lact 2006 22 2 188 194 10.1177/0890334406287148 16684907

[B26] Prasad B Costello AMdeL Impact and sustainability of a 'baby friendly' health education intervention at a district hospital in Bihar, India BMJ 1995 310 621 623 7703747 10.1136/bmj.310.6980.621 PMC2549005

[B27] Valdés V Schooley J The role of education in breastfeeding success Food and Nutrition Bulletin 1996 17 4 431 437

[B28] Davies-Adetugbo AA Fabiyi AK Ojoofeitimi EO Adetugbo K Breastfeeding training improves health worker performance in rural Nigeria East Afr Med J 1997 74 8 510 513 9487417

[B29] Moussa Abba A De Koninck M Hamelin A-M Obstacles to exclusive breastfeeding promotion in healthcare facilities in the city of Niamey, Niger: health professionals' opinions J Hum Lact in press

[B30] Mayer R Deslauriers JP Mayer R, Ouellet F, Saint-Jacques M-C, Turcotte D et collaborateurs Quelques éléments d'analyse qualitative: l'analyse de contenu, l'analyse ancrée, l'induction analytique et le récit de vie Méthodes de Recherche en Intervention Sociale 2000 Gaëtan Morin Éditeur. Montréal, Québec 159 189

[B31] Jaffré Y Olivier de Sardan J-P Les Dysfonctionnements des Systèmes de Soins. Rapport du Volet Socio-Anthropologique. Enquêtes sur l'Accès aux Soins dans 5 Capitales d'Afrique de l'Ouest Projet "santé urbaine" (UNICEF-Coopération française) 2002

[B32] WHO (World Health Organization) The International Code of Marketing of Breastmilk Substitutes 1981 Geneva: WHO 10.1001/archpedi.1981.02130340004002 7293987

[B33] Institut National de la Statistique (INS), Macro International Inc Enquête Démographique et de Santé et à Indicateurs Multiples du Niger 2006 2007 INS et Macro International Inc., Claverton, Maryland, USA

[B34] UNICEF, Ministère de la Santé publique-Niger Initiative Hôpitaux Amis des Bébés au Niger. Protéger, Promouvoir, Soutenir l'Allaitement Maternel. Evaluation du Niveau de Mise en œuvre Niger 2007

[B35] Dillaway HE Douma ME Are pediatric offices "supportive" of breastfeeding? Discrepancies between mothers' and healthcare professionals' reports Clin Pediatr (Phila) 2004 43 417 430 10.1177/000992280404300502 15208746

[B36] OMS (Organisation Mondiale de la Santé) Données Scientifiques Relatives aux Dix Conditions pour le Succès de l'Allaitement 1999 Genève: Département santé et développement de l'enfant et de l'adolescent

[B37] OMS/UNICEF (Organisation Mondiale de la Santé/Le Fonds des Nations-Unies pour l'Enfance) Protection, Encouragement et Soutien de L'Allaitement Maternel: Le Rôle Spécial des Services Liés à la Maternité. Déclaration Conjointe de l'OMS et de l'UNICEF 1992 Genève: OMS

[B38] American Academy of Pediatrics Breastfeeding and the use of human milk: work group on breastfeeding Pediatrics 1997 110 6 1035 1039 10.1542/peds.100.6.1035 9411381

[B39] WHO (World Health Organization) Complementary Feeding of Young Children in Developing Countries: a Review of Current Scientific Knowledge 1998 Geneva: WHO

[B40] AMS (Assemblée Mondiale de la Santé) La Nutrition chez le Nourrisson et le Jeune Enfant. 54e Assemblée Mondiale de la Santé, Résolution WHA 54.2 Genève 2001

